# Maternal *H. pylori* seropositivity is associated with gestational hypertension but is irrelevant to fetal growth and development in early childhood

**DOI:** 10.1186/s12887-019-1863-2

**Published:** 2019-12-16

**Authors:** Fu-Ping Lai, Yi-Fang Tu, Bor-Shyang Sheu, Yao-Jong Yang

**Affiliations:** 1 Departments of Pediatrics, National Cheng Kung University Hospital, Medical College, National Cheng Kung University, 138 Sheng Li Rd, Tainan, 70428 Taiwan; 2 Internal Medicine, National Cheng Kung University Hospital, Medical College, National Cheng Kung University, Tainan, Taiwan; 3 Institutes of Clinical Medicine, National Cheng Kung University Hospital, Medical College, National Cheng Kung University, Tainan, Taiwan

**Keywords:** Growth and development, Ghrelin, *H. pylori*, Insulin-like growth factor-1, Gestational hypertension, Child, Pregnancy

## Abstract

**Background:**

*Helicobacter pylori* infection is known to alter growth-related hormones and affect growth in young children. However, it is still unknown whether maternal *H. pylori* infection has an impact on the levels of cord blood growth-related hormones and whether this can predict intrauterine growth restriction and poor physical and neurodevelopmental outcomes in children. This study aimed to examine associations between maternal *H. pylori* infection and pregnancy-related adverse events, fetal growth and early childhood development.

**Methods:**

In this prospective cohort study, we recruited singleton pregnant women without major medical illnesses from January 2014 to January 2015. Seropositivity for *H. pylori* was defined as > 12 U/ml of anti-*H. pylori* IgG in maternal serum. Demographic data and pregnancy-related medical issues of the cohort were documented. Cord blood levels of insulin-like growth factor-1 (IGF-1), insulin-like growth factor binding protein-3 (IGFBP-3), insulin, and ghrelin were determined using ELISA. The growth of the included neonates was monitored annually for up to 3 years, and cognitive development was assessed using the comprehensive developmental inventory for infants and toddlers (CDIIT) test 3 years after birth.

**Results:**

Of the 106 enrolled women, 25 (23.6%) were *H. pylori*-seropositive. Maternal *H. pylori* seropositivity was correlated with a higher risk of developing gestational hypertension (GH) (12% vs. 1.2%, *p* = 0.04) and lower cord blood levels of IGF-1 (< 35 ng/ml, 70.0% vs. 40.7%, *p* = 0.02) and IGFBP-3 (< 1120 ng/ml, 100.0% vs. 76.3%, *p* = 0.02) compared with the seronegative women. No significant impacts on birth weight, childhood growth and cognitive development were found to be correlated with maternal *H. pylori* seropositivity during pregnancy.

**Conclusions:**

Maternal *H. pylori* infection during pregnancy was more likely to lead to the development of GH, but was not correlated with fetal and childhood growth and development. In addition to close monitoring of hypertension, *H. pylori* eradication can be considered for mothers with *H. pylori* infection.

## Background

*Helicobacter pylori* infects more than half of the global population, although its prevalence varies widely among different countries. Low socioeconomic status and poor sanitary or hygienic conditions are associated with the prevalence of *H. pylori* infection. Primary *H. pylori* infections occur most commonly in early childhood, with reported annual spontaneous seroreversion rates ranging from 1 to 2% both in children and adults [1, 2]. Although it seldom causes clinical symptoms in children, chronic *H. pylori* infection can pose serious health threats, and the bacterium has been reported to promote the development of chronic gastritis, peptic ulcer diseases, MALT lymphoma and gastric cancer in 10% of the infected population. Furthermore, there is growing evidence, mainly obtained from observational studies, showing that *H. pylori* infection may impair growth in children [3–5]. *H. pylori*-induced chronic gastritis results in the loss of appetite, malabsorption of nutrients, and dysregulation of the gastric endocrine and growth hormone systems, all of which may contribute to childhood growth impairment [6]. We previously showed that the successful eradication of *H. pylori* can restore systemic ghrelin levels and improve growth in children [7].

*H. pylori* infection during pregnancy has been associated with several adverse outcomes in both mothers and neonates [8, 9]. Two cohort studies conducted in Uganda and Sudan demonstrated that maternal *H. pylori* infection was correlated with a low neonatal birth weight [10, 11], however this effect was not observed in mouse models [12]. Another prospective cohort study conducted in the Netherlands identified *H. pylori* infection as an independent risk factor for frequent vomiting during pregnancy, and that this was correlated with an increase in the incidence of small for gestational age (SGA) neonates [8]. Moreover, a separate case-control study revealed that a significantly higher percentage of women positive for *H. pylori* stool antigen (HPSA) (indicative of *H. pylori* infection) developed preeclampsia (PE) with intrauterine growth restriction (IUGR) compared with HPSA-negative women. It is thought that *H. pylori*-induced iron deficiency anemia (IDA) also plays a role in driving IUGR [13, 14]. These results indicate possible etiopathological connections between maternal *H. pylori* infection and IUGR. It has previously been documented that cord blood levels of insulin, insulin-like growth factors (IGFs), insulin-like growth factor binding proteins (IGFBPs), and ghrelin are correlated with intrauterine fetal growth [15–18]. However, no previous study has addressed the role of these growth factors and hormones in maternal *H. pylori* infection and IUGR.

Children born SGA are associated with poor neurodevelopmental outcomes [19–21]. Similarly, *H. pylori* infection has been negatively correlated with cognitive development in children of early school age [22]. Interestingly, intraperitoneal injections of *H. pylori* filtrate have been shown to be sufficient to induce spatial learning and memory deficits in rats [23]. However, it is currently unclear whether maternal *H. pylori* infection has a negative impact on the neurodevelopment potential of the fetus. In this prospective cohort study, we investigated the effects of maternal *H. pylori* infection and related pregnancy disorders on the growth and development of fetuses, neonates, and during early childhood.

## Methods

### Subject recruitment and follow-up

Singleton pregnant women who attended regular antenatal examinations at one obstetric-pediatric clinic in Tainan City, Taiwan, between January 2014 and January 2015 were identified and recruited into this study. Eligibility was then assessed between 28 and 32 weeks of gestation. Individuals with underlying medical conditions such as chronic hypertension, pre-gestational diabetes mellitus, chronic lung disease, renal disease, major cardiac disease, autoimmune conditions, thyroid disease, malignancy, and uterine malformations were excluded. Individuals that had a history of illicit drug abuse and those whose fetuses had chromosomal abnormalities, congenital malformations or evident congenital infections (TORCH) were also excluded. Follow-up assessments were carried out at the time of delivery, and at 1, 2, and 3 years after delivery.

This study was approved by the Ethics Committee (B-BR102–001) of National Cheng Kung University Hospital, Tainan, Taiwan, and written informed consent was obtained from each participant and her spouse. The demographic characteristics, anthropometric data, and common risk factors of SGA were collected and assessed, including maternal age, body height, body weight before pregnancy, body mass index (BMI) before pregnancy, smoking tobacco, alcohol use, maternal educational attainment, annual household income, and pregnancy complications such as antepartum bleeding, anemia, pregnancy-induced hypertension (PIH), and PE. Anemia was defined as a hemoglobin concentration of < 11 g/dL. PIH was defined as any new onset of hypertension (systolic blood pressure ≥ 140 mmHg and/or diastolic blood pressure ≥ 90 mmHg) after 20 weeks of gestation. PE was defined as a combination of PIH and proteinuria or signs of end-organ dysfunction.

The corresponding neonates enrolled in the follow-up were full-term (gestational age 37–40 weeks) and healthy. Parameters recorded at birth included gestational age, body weight and length, head circumference, and Apgar score at 1 and 5 min post-delivery. Neonates that required post-delivery intensive care were excluded from the follow-up study.

### Maternal serum collection and testing for anti-*H. pylori* IgG

The status of *H. pylori* infection was assessed by measuring serum IgG against *H. pylori* using a commercial *H. pylori* IgG ELISA kit (IBL, Hamburg, Germany) at 28–32 weeks of gestation, the period when a routine screening test for hepatitis B surface antigen is commonly conducted in Taiwan. Anti-*H. pylori* IgG titers > 12 U/ml were considered to be positive, while titers < 8 U/ml were considered to be negative. Titer values between 8 and 12 U/ml were considered to be equivocal. The mothers were subsequently categorized as being either *H. pylori*-seropositive or *H. pylori*-seronegative according to the ELISA results.

### Cord blood levels of IGF-1, IGFBP-3, insulin, and ghrelin

Cord venous blood samples were collected at delivery and centrifuged at 3500 x g for 30 min at 4 °C to separate the serum. The serum samples were stored at − 80 °C. IGF-I (R&D Systems, Inc. Minneapolis, MN, USA), IGFBP-3 (R&D Systems, Inc. Minneapolis, MN, USA), insulin ((R&D Systems, Inc. Minneapolis, MN, USA) and ghrelin (EMD Millipore Corporation, St. Charles, MO, USA) levels were measured using ELISA following the manufacturers’ instructions.

### Assessment of anthropometric parameters and cognitive development of newborns

The enrolled newborns were studied longitudinally for up to 3 years. The weight and length of each child were measured at birth and then annually. According to the gestational age of infants born in Taiwan, SGA was defined by a birth weight below the 10th percentile [24].

Cognitive development was assessed using the comprehensive developmental inventory for infants and toddlers (CDIIT) test at 3 years of age. The CDIIT is a reliable pediatric norm-referenced assessment tool that is widely used for the clinical diagnosis of developmental delays in five major developmental areas, including cognition, language, motor, social and self-care skills [25, 26]. The CDIIT test consists of a diagnostic test (CDIIT-DT) and a screening test (CDIIT-ST). In this study, we applied the cognition subtest of the CDIIT-DT and assessed five aspects of a child’s mental capacity, including attention, perception, memory, reasoning and concepts of color, shape, size, and number. The evaluations were conducted by a trained administrator.

### *H. pylori* stool antigen test (HPSA) and definition of *H. pylori* infection in children

Stool samples were collected from the enrolled infants at 1, 2 and 3 years after birth to detect new *H. pylori* infections using the HPSA test. The HPSA test (Meridian Diagnostic Inc., Cincinnati, Ohio, USA) uses a plurality of monoclonal anti-*H. pylori* antibodies adsorbed to microwells. The results were interpreted spectrophotometrically, and the cutoff optical density at 450 nm for a positive outcome was set at 0.14. Children with a positive HPSA test in any one of the three samples were considered to be infected with *H. pylori*, while those who had a negative HPSA test result following a previous positive result were defined as having spontaneous elimination of *H. pylori* infection. A minimum of two consecutive positive HPSA tests during the follow-up period was considered to indicate persistent *H. pylori* infection. Children with negative HPSA tests throughout the follow-up period were considered to be non-infected.

### Statistical analysis

Demographic data and measurable parameters were presented as frequencies and means ± standard deviations (SDs). Significance of association was determined using the Pearson chi-square (χ2) test for categorical variables and the independent sample *t*-test for continuous variables. As ELISA tests tend to produce high SD values which may give rise to type II statistical errors, receiver operating characteristic (ROC) curve analysis in conjunction with Youden’s index was used to determine the best cutoff values of cord blood IGF-1, IGFBP-3, insulin and ghrelin levels to differentiate *H. pylori*-seropositive and *H. pylori*-seronegative mothers. A *p* value of less than 0.05 was considered to be statistically significant. All statistical analyses were performed using SPSS Statistics V.17.0.

## Results

### Study design and enrolled subjects

**Fig. 1:**
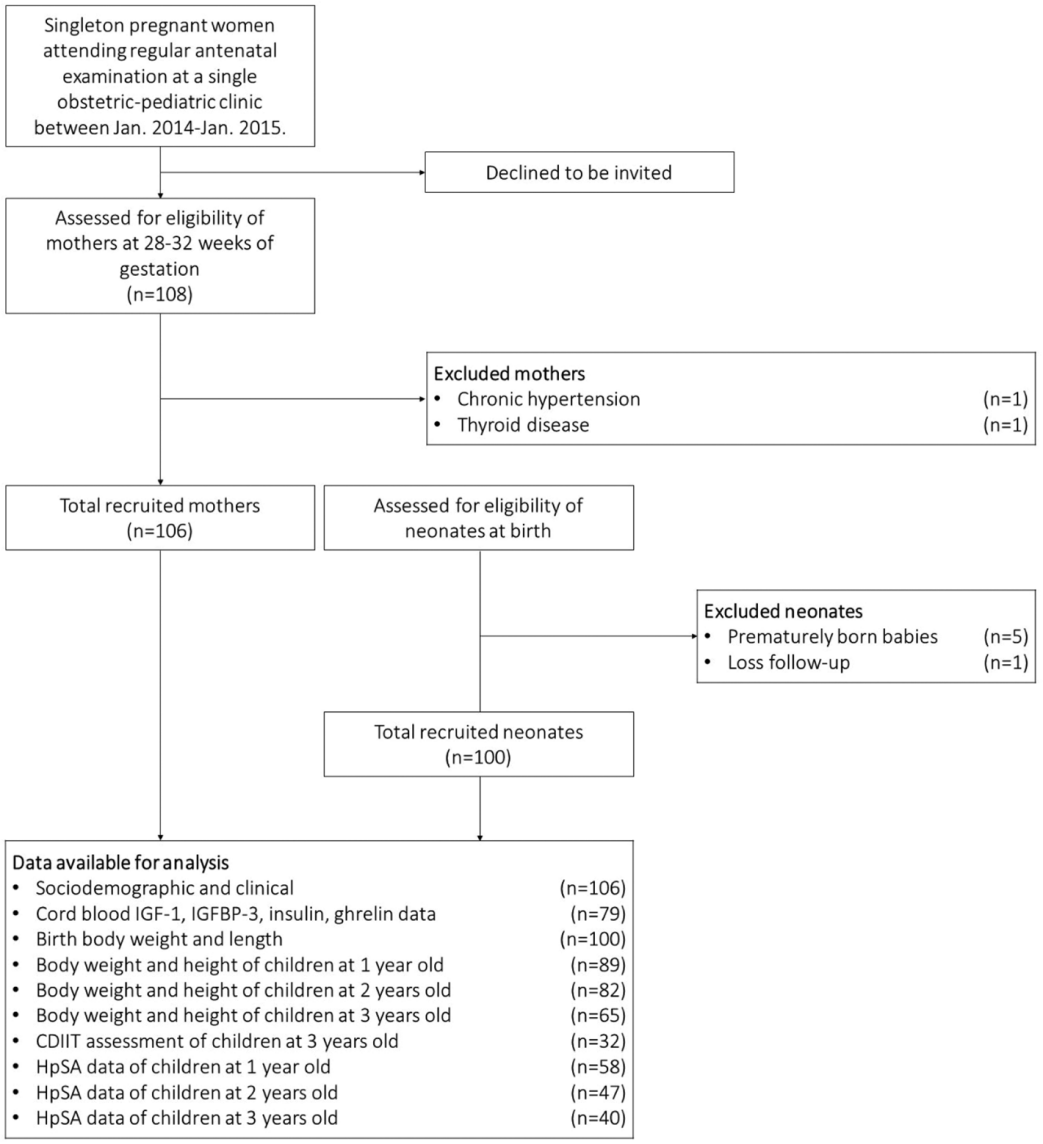
The workflow and number of cases of the study

Figure 1 shows the workflow of the study and the data collected. A total of 108 singleton pregnant women were initially recruited. Two participants were eventually excluded due to chronic hypertension and thyroid disease. The sera of the remaining 106 participants were collected and tested for anti-*H. pylori* IgG as specified. A total of 79 cord blood samples were analyzed for IGF-1, IGFBP-3, insulin, and ghrelin levels. For the follow-up assessments, five preterm newborns (gestational age < 37 weeks) and one who was lost to follow-up were removed from the cohort.

### Seropositivity and clinical characteristics of the mothers

Twenty-five (23.6%) of the 106 pregnant women were positive for serum anti-*H. pylori* IgG (Table 1). There were no significant differences in age, pre-pregnancy body weight, height, BMI, household size, annual household income, educational attainment, job, smoking habit, alcohol drinking habit, hemoglobin, and placental weight between the two groups (*p* > 0.05). A significantly higher incidence rate of PIH was observed in the *H. pylori-*seropositive mothers than in the seronegative mothers (12% vs 1.2%, *p* < 0.05). In addition, the cord blood IGF-1 and IGFBP-3 levels were modestly lower in the *H. pylori*-seropositive group compared with the seronegative group. According to our ROC curve analysis and Youden’s index, the *H. pylori*-seropositive mothers were significantly more likely to have a cord blood IGF-1 level < 35 ng/ml (70.0% vs 40.7%, *p* = 0.023) and IGFBP-3 level < 1250 ng/ml (100% vs 76.3%, *p* = 0.02) compared with the seronegative mothers (Table 2).

**Table 1:** The characteristics of the mothers in the *H. pylori*-seropositive and seronegative groups

Variable, mean ± SD or number (%)	Serum *H. pylori* IgG		*p* value
	Seropositive (*n* = 25)	Seronegative (*n* = 81)	
Maternal age (years)	31.0 ± 4.0	32.1 ± 3.8	0.20
Maternal body height (cm)	159.5 ± 5.5	159.9 ± 4.4	0.70
Maternal pre-pregnancy body weight (kg)	52.2 ± 5.9	55.5 ± 8.2	0.06
Maternal pre-pregnancy BMI (kg/m^2^)	20.6 ± 2.5	21.7 ± 3.1	0.09
Household size (person)	3.8 ± 1.7	3.7 ± 1.8	0.69
Annual household income			0.79
Low^a^	7 (28.0)	25 (30.9)	
Middle/High	18 (72.0)	56 (69.1)	
Educational attainment			0.68
Low/Middle^b^	1 (4.0)	8 (11.1)	
High	24 (96.0)	73 (67.9)	
Housewife	5 (20.0)	25 (30.9)	0.45
Primigravida	18 (72.0)	48 (59.3)	0.25
Smoking	0 (0.0)	1 (1.2)	1.00
Alcohol	3 (12.0)	5 (6.2)	0.39
Gestational age (weeks)	38.5 ± 1.0	38.8 ± 1.1	0.26
Placental weight (g)	505.2 ± 65.3	508.5 ± 47.4	0.79
Hemoglobin (g/dL)	11.9 ± 1.7	11.9 ± 1.4	0.98
Pregnancy-induced hypertension	3 (12.0)	1 (1.2)	0.04
Preeclampsia	0 (0.0)	0 (0.0)	1.00
Bleeding at early gestation	0 (0.0)	2 (2.5)	1.00

^a^Annual household income < 16,200 USD

^b^Educational attainment below bachelor level

**Table 2:** Associations between cord blood IGF-1, IGFBP-3, ghrelin, and insulin levels and maternal *H. pylori* seropositivity

Variable	Serum *H. pylori* IgG		*p* value
Number (%)	Seropositive (*n* = 20)	Seronegative (*n* = 59)	
IGF-1 < 35 (ng/ml)	14 (70.0)	24 (40.7)	0.02
IGFBP-3 < 1120 (ng/ml)	20 (100)	45 (76.3)	0.02
Insulin < 35 (pmol/l)	17 (85.0)	38 (64.4)	0.10
Ghrelin > 925 (pg/ml)	3 (15.0)	16 (27.1)	0.37

### Associations between maternal *H. pylori* seropositivity and birth weight, early childhood growth, and cognitive development

Table 3 shows the anthropometric data and cognitive development of the children with *H. pylori*-seropositive and seronegative mothers in the first 3 years after birth. No significant differences in body weight, length and head circumference at birth were observed between the *H. pylori-*seropositive group and seronegative group. Likewise, the rates of SGA and low birth weight (birth body weight < 2500 g) were similar in the two groups. There were also no significant differences in body weight and height subsequently measured at 1, 2, and 3 years after birth between the children born to *H. pylori*-seropositive and seronegative mothers. Lastly, CDIIT assessments of the children at 3 years of age revealed no significant difference in cognitive development between the two groups of children (Table 3).

**Table 3:** The anthropometric characteristics and cognitive development of the children by maternal *H. pylori* status

Variable	Serum *H. pylori* IgG		*p* value
Mean ± SD or number (%)	Seropositive (*n* = 23)	Seronegative (*n* = 77)	
Birth body length (cm)	50.0 ± 1.5	50.1 ± 2.1	0.70
Birth head circumference (cm)	33.8 ± 1.3	33.9 ± 1.4	0.68
Birth body weight (g)	3012.7 ± 375.3	3118.7 ± 391.4	0.25
Low birth weight < 2500 g	2 (8.7)	4 (5.2)	0.62
Small for gestational age (SGA)	2 (8.7)	8 (10.4)	1.00
Body weight (kg)			
1 year of age	9.0 ± 1.0 (n = 23)	9.2 ± 1.1 (*n* = 66)	0.47
2 years of age	11.8 ± 1.3 (*n* = 22)	12.2 ± 1.4 (*n* = 60)	0.29
3 years of age	13.8 ± 1.6 (n = 20)	14.5 ± 2.1 (n = 45)	0.18
Body height (cm)			
1 year of age	75.4 ± 2.4 (n = 23)	74.7 ± 2.6 (n = 66)	0.24
2 years of age	86.4 ± 2.8 (n = 22)	87.1 ± 3.6 (*n* = 60)	0.45
3 years of age	94.0 ± 3.7 (n = 20)	95.6 ± 4.7 (*n* = 45)	0.19
Domains of the CDIIT test	(*n* = 14)	(*n* = 18)	
Attention	109.3 ± 16.9	108.2 ± 18.9	0.87
Perception	106.9 ± 13.6	106.4 ± 15.2	0.93
Memory	106.4 ± 22.9	107.6 ± 23.9	0.89
Reasoning	110.1 ± 15.3	110.8 ± 17.1	0.91
Concepts	105.3 ± 16.2	103.5 ± 20.9	0.79

### Susceptibility to *H. pylori* infection in the children during the follow-up period

Serial HPSA tests were performed in the children at 1, 2, and 3 years after birth. None of the 58 children who received HPSA tests were infected with *H. pylori* in the first year. In the second year, two of 16 children (12.5%) from the *H. pylori*-seropositive group and one of 31 children (3.2%) from the *H. pylori-*seronegative group were found to be HPSA positive (*p* = 0.26). In the third year, one more child from the maternal *H. pylori*-seropositive group was found to be HSPA positive. However, one child that was previously HSPA positive became HPSA negative, while two children remained HSPA positive (persistent infection).

### Analysis of the risk factors for SGA in singleton term neonates

The risk factors for SGA were further evaluated (Table 4). There were no significant differences in maternal anthropometric and socio-demographic characteristics between the SGA and non-SGA groups. However, the SGA group exhibited a significantly lower placental weight (445.0 vs 514.9 g, *p* < 0.01), lower cord blood IGF-1 level (24.7 vs 40.1 ng/mL, *p* = 0.04) and higher ghrelin level (1045.1 vs 782.3 pg/mL, *p* < 0.01) compared with the non-SGA group.

**Table 4:** The risk factors for SGA using univariate analysis

Variable, Mean ± SD	SGA	Non-SGA	*p* value
Placental weight (g)	445.0 ± 47.9 (*n* = 10)	514.9 ± 47.4 (*n* = 90)	< 0.01
IGF-1 (ng/mL)	24.7 ± 10.5 (n = 7)	40.1 ± 18.8 (n = 69)	0.04
IGFBP-3 (ng/mL)	834.5 ± 363.7 (n = 7)	759.1 ± 323.8 n (*n* = 69)	0.56
Insulin (pmol/L)	22.7 ± 17.9 (n = 7)	29.9 ± 29.3 (n = 69)	0.53
Ghrelin (pg/mL)	1045.1 ± 241.6 (n = 7)	774.3 ± 195.8 (n = 69)	< 0.01

SGA, small for gestational age

## Discussion

This is the first 3-year prospective cohort study to evaluate the relevant implications of maternal *H. pylori* infection during pregnancy on fetal growth, as well as growth and cognitive development in young children. Our results showed that the *H. pylori*-seropositive mothers had a higher risk of developing PIH during pregnancy. In addition, the *H. pylori*-seropositive mothers had lower levels of cord blood IGF-1 and IGFBP-3 than the seronegative mothers, even though no apparent changes in birth weight and neonatal size were observed. Our results suggested that early childhood growth and cognitive development were not affected by maternal *H. pylori* infection during pregnancy.

In contrast to our observations, a previous study conducted by Eslick et al. reported that *H. pylori*-seropositive mothers were more likely to give birth to undersized neonates than seronegative women due to IUGR [27]. In a separate study conducted in Uganda, Wanyama et al. showed that maternal *H. pylori* infection was an independent predictor of low birth weight in newborns [10]. Likewise, Mustafa et al. also demonstrated that maternal *H. pylori* seropositivity was more frequently associated with low birth weight [11]. However, these studies did not consider the effects of other potential confounding events that are commonly experienced during pregnancy, such as severe nausea, vomiting, PE, and anemia. We hypothesize that these factors could potentially explain the discrepant findings between previous studies and the current study.

Although not entirely understood, the mechanism by which maternal *H. pylori* infection impacts birth weight may be multifactorial. Maternal *H. pylori* infection has been reported to be a risk factor for hyperemesis gravidarum, PE, and IDA during pregnancy [8, 13, 28–30], and these events also contribute to SGA or IUGR. Interestingly, virulent factors of *H. pylori* have also been considered to be a cause of IUGR. Similarly, previous studies have reported that persistent CagA/VacA-positive *H. pylori* infection in pregnant women can cause PE and IUGR [31], and that SGA was correlated specifically with infections caused by CagA-positive strains of *H. pylori* [32]. Although the virulence factors of *H. pylori* from infected mothers were not tested in the current study, almost all strains of *H. pylori* isolated from Taiwanese patients are CagA/VacA-positive [33]. Notably, the rate of PIH was higher in the *H. pylori-*seropositive group compared with the seronegative group. However, no significant correlations were observed for anemia or preeclampsia.

Since *H. pylori* infection has been reported to alter growth hormones [32], we investigated the effects of *H. pylori* infection on cord blood levels of IGF-1, IGFBP-3, insulin, and ghrelin, as well as the relationships between these hormones and IUGR. Consistent with previous studies which reported decreased levels of IGF-1, IGFBP-3, and insulin, and increased levels of ghrelin in the cord blood of IUGR neonates [17, 34–36], our data showed significantly lower levels of IGF-1 and higher levels of ghrelin in the cord blood samples from the SGA neonates compared with those from the non-SGA neonates. Our results also revealed that maternal *H. pylori*-seropositivity during pregnancy was correlated with lower levels of IGF-1 and IGFBP-3 in the cord blood. However, of all the potential risk factors and parameters considered, only placental weight, but not PIH or *H. pylori*-seropositivity, was found to be associated with SGA.

Taken together, it is likely that *H. pylori* infection during pregnancy causes SGA via indirect mechanisms such as the aforementioned adverse effects that are commonly associated with *H. pylori* infection. However, further studies are required to confirm this.

The data obtained in the current study indicated that there were no significant differences in early childhood growth and cognitive development between the children born to *H. pylori*-seropositive mothers and seronegative mothers. In addition, maternal *H. pylori*-seropositivity during pregnancy did not increase the risk of acquiring *H. pylori* infection in children. This is consistent with our observations that maternal *H. pylori* infection status did not affect initial birth weight.

There are several limitations to this study. First, SGA was used as a surrogate for IUGR. However, this clinical definition does not distinguish between constitutionally and pathologically small fetuses [37, 38]. On the other hand, although suffering from intrauterine growth deceleration, IUGR infants may have appropriate birth weight as per gestation. “True” IUGR infants are mostly a consequence of placental insufficiency, and they present with poorer perinatal and long-term outcomes compared with constitutionally SGA neonates [38, 39]. Thus, ways to more effectively distinguish neonates with IUGR would be more clinically relevant. Second, not all risk factors of SGA were considered in this study. SGA risk factors such as maternal weight gain, nutritional status during pregnancy, PE and the prevalence and severity of hyperemesis gravidarum may also play important roles and should therefore be studied in the future. Indeed, maternal weight gain during pregnancy has been shown to be positively correlated with neonatal birth weight [40]. Third, given that placental weight was negatively correlated with the risk of SGA [41, 42], we relied on placental weight as a representative of overall placental condition. However, more in-depth evaluations of specific placental parameters such as uterine artery velocimetry or expression of biomarkers should be performed. This would allow important weight-independent physiological and pathological changes of the placenta to be detected more effectively. Furthermore, our serology *H. pylori* IgG test, which was used to define infection status in our cohort, did not distinguish previously cleared infections from ongoing infections [43]. Lastly, there were missing data during the 3-year follow-up period, which may have led to bias in the results.

## Conclusions

In this prospective cohort study in Taiwan, we found that maternal *H. pylori* infection per se did not promote SGA in neonates. We showed that SGA was most likely caused by other *H. pylori*-induced pathologies and pregnancy-related complications such as hyperemesis gravidarum, PE, and anemia. Moreover, we revealed that maternal *H. pylori* infection did not directly impair growth and cognitive development during early childhood. However, as *H. pylori*-infected pregnant women are more likely to develop PIH, increased attention should be paid to prevent hypertension-related complications in these individuals. Future studies to investigate the possible mechanisms by which *H. pylori* directly affects PIH and to assess whether *H. pylori* eradication can decrease the risk of PIH for mothers are warranted.

## Data Availability

The datasets used and analyzed during the current study are available from the corresponding author on reasonable request.

## Abbreviations

BMI: body mass index
CDIIT: comprehensive developmental inventory for infants and toddlers
GH: gestational hypertension
HPSA: *H. pylori* stool antigen
IDA: iron deficiency anemia
IGFBPs: insulin-like growth factors binding proteins
IGFs: insulin-like growth factors
IUGR: intrauterine growth restriction
PE: preeclampsia
SGA: small for gestational age
